# Hypertension in a rural community in South Africa: what they know, what they think they know and what they recommend

**DOI:** 10.1186/s12889-019-6642-3

**Published:** 2019-03-25

**Authors:** Vita W. Jongen, Samanta T. Lalla-Edward, Alinda G. Vos, Noortje G. Godijk, Hugo Tempelman, Diederick E. Grobbee, Walter Devillé, Kerstin Klipstein-Grobusch

**Affiliations:** 1 Julius Global Health, Julius Center for Health Sciences and Primary Care, University Medical Center Utrecht, Utrecht University, PO Box 85500, Utrecht, The Netherlands; 2 Wits Reproductive Health and HIV Institute, University of the Witwatersrand, Faculty of Health Sciences, Johannesburg, South Africa; 3 Department of Internal Medicine, University Medical Center Utrecht, Utrecht, The Netherlands; 4 Ndlovu Care Group, Elandsdoorn, South Africa; 5 Division of Epidemiology and Biostatistics, School of Public Health, Faculty of Health Sciences, University of the Witwatersrand, Johannesburg, South Africa

**Keywords:** Hypertension, Hypertension perception, Hypertension knowledge, Rural community, Focus group discussions, South Africa

## Abstract

**Background:**

Hypertension is one of the most important risk factors for cardiovascular disease and has a high prevalence in South Africa and other low- and middle-income countries. However, awareness of hypertension has been reported to be low. Health programmes can increase awareness of hypertension and its causes, but hinge on the knowledge and perception of the targeted community. Therefore, this study investigated knowledge on and perceptions about hypertension of community members in a rural area in Limpopo, South Africa with the aim to increase awareness of hypertension and cardiovascular disease in the local population.

**Methods:**

Using a mixed methods study approach, 451 participants of the Ndlovu Cohort Study, attending a follow-up visit between August 2017 and January 2018, completed a questionnaire on cardiovascular risk perception. A knowledge score was calculated for all participants. Sixty participants were invited to participate in six focus group discussions, of which 56 participated. Audio recordings were transcribed verbatim, transcripts coded, and thematic analysis of the data undertaken to obtain an understanding of knowledge and perception of hypertension in the community.

**Results:**

Most members of the community seemed to have intermediate (74.3%) or good (14.0%) knowledge of hypertension based on the knowledge score, and only 11.8% of the population had poor knowledge. The risk factors of hypertension seemed to be well known in the community. Poverty was identified as a major vulnerability in this community limiting choices for healthy lifestyles such as nutritious foods, recreational physical activity and accessing health care timely. Participants proposed community-based activities as an effective way to reach out to community members for prevention and management of hypertension.

**Conclusion:**

This study highlights the need for improved health promotion efforts to increase knowledge of hypertension in rural communities, and to address poverty as a major obstacle to healthy life-style choices.

**Electronic supplementary material:**

The online version of this article (10.1186/s12889-019-6642-3) contains supplementary material, which is available to authorized users.

## Background

Non-communicable diseases (NCD) are currently accounting globally for 70% of all deaths. Most of these deaths (17.7 million) are due to cardiovascular diseases, such as myocardial infarction and stroke [1]. Hypertension is one of the most potent risk factors for cardiovascular disease (CVD), with over 1 billion people affected by it worldwide [2]. According to WHO data, approximately 27.4% of men and 26.1% of women in South Africa have hypertension [3], though prevalence of up to 60% has been reported [4].

Despite the high prevalence of hypertension in South Africa, both awareness and control are low among hypertensive individuals, with figures ranging between 19.0–56.0% and 4.0–33.0%, respectively [5–7]. In comparison, in high-income countries awareness and control of hypertension among hypertensive individuals has been reported to be as high as 82 and 51% respectively [8]. The low awareness- and control rates in South Africa are of concern as awareness and subsequent blood pressure control have the potential to significantly decrease both morbidity and mortality due to CVD [9].

The risk factors of hypertension are multifactorial and include being overweight, excessive salt intake, alcohol consumption, and lack of physical activity. Hypertension control can in part be reached by changes in lifestyle, such as dietary changes, e.g. reduction of salt intake, exercising to support weight reduction and by use of antihypertensive medication [10–12].

Better knowledge of hypertension has been reported to improve compliance to antihypertensive treatment and consequently the control of the disease [13, 14]. Successful implementation of a health program hinges on the existing knowledge and perception of the targeted community. Only a few studies in South Africa have focused on the knowledge that community members have on CVD, and even fewer on hypertension knowledge [15–18].

Based on the high prevalence of hypertension, generally low awareness levels and the lack of available South African data hereon, this study focused on the knowledge on and perceptions about hypertension of community members in a rural area in South Africa to improve community-based preventive strategies and management of hypertension. The following three research questions guided the study:

1. What do the community members know about hypertension?
2. Do community members regard hypertension as a health problem?
3. How would they recommend awareness should be raised about hypertension?

## Methods

A mixed method design, comprising of quantitative and qualitative methods, was used to conduct the study.

### Setting

This study was embedded in the Ndlovu cohort study (NCS) [19] at the Ndlovu Research Centre in Elandsdoorn, a township in the Moutse area in Limpopo, South Africa. Inclusion criteria for the NCS were age of 18 years or older and living in proximity of the research site. The current study was undertaken from August 2017 until January 2018. All participants attending a follow-up visit for the NCS during these months were invited to participate.

### Questionnaire on cardiovascular risk perception

Participants were asked to complete a questionnaire on cardiovascular risk perception, consisting of nine questions regarding awareness and perception of hypertension and cardiovascular diseases (see Additional file 1). The questionnaire was piloted prior to the start of data collection and was either administered in English or one of the local languages (Sepedi, isiZulu) by one of the counsellors of the NCS. Knowledge on hypertension was assessed by five of the nine questions and covered the risks, symptoms and influencing factors of hypertension.

### Focus group discussions on knowledge on and awareness of hypertension

A total of 60 participants were invited to participate in focus group discussions (FGD), taking place on two separate days in October 2017. Participants were all part of the NCS, but not necessarily part of the quantitative part of this study. Participants were recruited either by phone or after a follow-up visit. Selection was done based on the proximity to the clinic and willingness to participate in additional studies within the NCS.

FGDs were conducted by two (one male and one female) experienced moderators using a semi-structured interview guide (Additional file 2). These moderators together with NCS study staff performed role-plays to facilitate familiarity with the contents of the guide prior to the FGDs. Six FGDs were held with an average of nine participants per group and conducted in either isiZulu or English. Of these, two were held with males only, two with females only, and two comprised both males and females (referred to as the mixed group). Participants could choose to participate in whichever FGD they felt most comfortable. During the course of the FGD participants were encouraged to raise other relevant topics. The FGDs took between 50 and 100 min and were audio recorded. An assistant was always present during the discussions to take notes and translate locally used words or vernacular.

### Data analysis

Quantitative data were analysed using descriptive statistics, expressing continuous variables as mean with their standard deviation (SD). Categorical data were presented as count data with percentages. A knowledge score was computed for all participants based on the two knowledge related questions of the questionnaire (i.e. do the following factors influence the risk of hypertension and identification of correct statements about hypertension (by selecting true or false options)). Each correct answer contributed one point to the individual knowledge score of a participant, with a maximum of 25 points. Participants were considered to have good knowledge of hypertension with a score of 21 and above, intermediate knowledge with a score from 13 to 20, and poor knowledge with a score below 12. Subgroup analysis was done to assess the relation of age, gender, education, and employment status with the knowledge score. All data was analysed using SPSS version 23 (IBM SPSS Statistics for Windows, Version 23.0. Armonk, NY: IBM Corp.).

The audio recordings of the FGDs were transcribed verbatim. Interviews that were conducted in isiZulu were translated into English and back translated by a second transcriber. All transcripts were quality checked (STLE). A codebook was developed by one researcher (VJ) and reviewed by another (STLE). Thereafter double coding (VJ and STLE) was done to check for inter-coder reliability prior to organising data into themes and subthemes.

## Results

### Socio-demographic information

In total, 451 participants were included in the quantitative analysis of the study. Slightly more females (*n* = 229) than males (*n* = 222) participated, aged on average 40.9, respectively 42.8 years. Average age of the 29 women and 27 men participating in the FDGs was 43.7 and 41.0 years respectively. In both the questionnaire and the FGD group most of the participants had secondary education and were single. The reported unemployment rate was high (74% in the questionnaire group and 86% in the FGD group). Socio-demographic characteristics for the questionnaire- and the FDG participants are shown in Table 1.

**Table 1:** Socio-demographic characteristics of questionnaire- and focus group discussion participants

	Questionnaire		Focus group discussions	
	Female (*n* = 229)	Male (*n* = 222)	Female (*n* = 29)	Male (*n* = 27)
Age (yr), mean (SD)	40.9 (11.6)	42.8 (12.3)	43.7 (10.2)	41.0 (12.1)
Age group (yr), *n* (%)				
18–24	16 (7.0)	15 (6.8)	0 (0.0)	3 (11.1)
25–39	89 (39.0)	78 (35.1)	10 (34.5)	8 (29.6)
40–49	79 (34.5)	51 (23.0)	11 (37.9)	8 (29.6)
50	45 (19.7)	78 (35.1)	8 (27.6)	8 (29.6)
Highest education level, *n* (%)				
None	10 (4.4)	4 (1.8)	1 (3.4)	0 (0.0)
Primary	35 (15.3)	48 (21.6)	8 (27.6)	7 (25.9)
Secondary	88 (38.4)	96 (43.2)	9 (31.0)	12 (44.4)
Matric	77 (33.6)	51 (23.0)	6 (20.7)	5 (18.5)
College	18 (7.9)	17 (7.7)	4 (13.8)	3 (11.1)
University	1 (0.4)	6 (2.7)	1 (3.4)	0 (0.0)
Marital status, *n* (%)				
Single	81 (35.4)	78 (35.1)	9 (31.0)	11 (40.7)
Married	50 (21.8)	61 (27.5)	6 (20.7)	4 (14.8)
Life Partner	42 (18.3)	45 (20.3)	7 (24.1)	8 (29.6)
Living together 50% of the time	28 (12.2)	19 (8.6)	3 (10.3)	2 (7.4)
Divorced	9 (3.9)	10 (4.5)	1 (3.4)	0 (0.0)
Widowed	19 (8.3)	8 (3.6)	3 (10.3)	2 (7.4)
Other	0 (0.0)	1 (0.5)	0 (0.0)	0 (0.0)
Employment status, *n* (%)				
Unemployed	177 (77.3)	158 (71.2)	26 (89.7)	22 (81.5)
Employed	52 (22.7)	64 (28.8)	3 (10.3)	5 (18.5)

Abbreviations: yr., year; SD, standard deviation

### Quantitative results

Results of the cardiovascular disease perception questionnaire were presented as adaptions of the actual questions (Table 2). For the original questionnaire see supplementary material (Additional file 1). Of the 451 participants 20.2% (*n* = 91) considered themselves to be informed very well about hypertension, whereas 19.1% (*n* = 86) considered themselves not to be informed at all. Factors that were mostly considered to influence the risk of hypertension were salt intake, stress, consumption of fast food and alcohol. About 90% of the participants were of the opinion that blood pressure can be improved by exercise and a healthy diet. Almost 90% reported that hypertension is bad for you, that it will make you feel sick, and that it increases the risk of a stroke.

**Table 2:** Questionnaire data regarding the awareness and perception of hypertension and CVD, categorized per gender

	Female (*n* = 229 (50.8%))			Male (*n* = 222 (49.2%))		
	yes	no	don’t know	yes	no	don’t know
Has family members with hypertension	124 (54.1)	103 (45.0)	2 (0.9)	69 (31.1)	145 (65.3)	8 (3.6)
Has acquaintances with hypertension	99 (43.2)	124 (54.1)	6 (2.6)	53 (23.9)	157 (70.7)	12 (5.4)
Risk factors associated with hypertension:						
Physical exercise	111 (48.5)	104 (45.4)	14 (6.1)	103 (46.4)	105 (47.3)	14 (6.3)
Smoking	137 (59.8)	59 (25.8)	33 (14.4)	169 (76.1)	34 (15.3)	19 (8.6)
Diet	126 (55.0)	89 (38.9)	14 (6.1)	114 (51.4)	82 (36.9)	26 (11.7)
Stress	205 (89.5)	23 (10.0)	1 (0.4)	175 (78.8)	34 (15.3)	13 (5.9)
Religion	45 (19.7)	156 (68.1)	28 (12.2)	30 (13.5)	163 (73.4)	29 (13.1)
Family History	137 (59.8)	62 (27.1)	30 (13.1)	115 (51.8)	75 (33.8)	32 (14.4)
Alcohol Consumption	151 (65.9)	44 (19.2)	34 (14.8)	168 (75.7)	32 (14.4)	22 (9.9)
Gender	54 (23.6)	144 (62.9)	31 (13.5)	46 (20.7)	148 (66.7)	28 (12.6)
The weather	101 (44.1)	105 (45.9)	23 (10.0)	103 (46.4)	96 (43.2)	23 (10.4)
Salt Intake	194 (84.7)	28 (12.2)	7 (3.1)	185 (83.3)	25 (11.3)	12 (5.4)
Pregnancy	165 (72.1)	46 (20.1)	18 (7.9)	96 (43.2)	60 (27.0)	66 (29.7)
Statements:						
Hypertension is bad for you	188 (82.1)	41 (17.9)	NA	189 (85.1)	33 (14.9)	NA
Fast food increases blood pressure	195 (85.2)	34 (14.8)	NA	176 (79.3)	46 (20.7)	NA
Salt increases blood pressure	206 (90.0)	23 (10.0)	NA	205 (92.3)	17 (7.7)	NA
Alcohol increases blood pressure	184 (80.3)	45 (19.7)	NA	195 (87.8)	27 (12.2)	NA
Blood pressure improves by a healthy diet	211 (92.1)	18 (7.9)	NA	200 (90.1)	22 (9.9)	NA
Blood pressure improves with exercise	213 (93.0)	16 (7.0)	NA	200 (90.1)	22 (9.9)	NA
Blood pressure can be managed by going to church	95 (41.5)	134 (58.5)	NA	84 (37.8)	138 (62.2)	NA
Blood pressure can be managed by praying	103 (45.0)	126 (55.0)	NA	93 (41.9)	129 (58.1)	NA
Blood pressure can be managed by a traditional healer	30 (13.1)	199 (86.9)	NA	33 (14.9)	189 (85.1)	NA
High blood pressure makes you feel sick	205 (89.5)	24 (10.5)	NA	198 (89.2)	24 (10.8)	NA
High blood pressure is a rare condition	80 (34.9)	149 (65.1)	NA	89 (40.1)	133 (59.9)	NA
High blood pressure prevents the development of heart disease	57 (24.9)	172 (75.1)	NA	66 (29.7)	156 (70.3)	NA
High blood pressure increases the risk of a stroke	215 (93.9)	14 (6.1)	NA	205 (92.3)	17 (7.7)	NA
If you ignore it, high blood pressure will go away on its own	69 (30.1)	160 (69.9)	NA	49 (22.1)	173 (77.9)	NA

Abbreviations: NA, not applicable

The average knowledge score of the 451 participants was 17.0 (SD 3.5). Most participants had intermediate knowledge of hypertension (74.3%), 14.0% of the participants had good knowledge, and 11.8% had poor knowledge. Knowledge statements with the lowest number of correct answers were that hypertension makes you feel sick (10.6% of answers were correct) and that gender influences the risk of hypertension (22.2% of answers were correct). Subgroup analysis showed no significant relationship between age, gender and employment status with knowledge score. Higher educational level was associated with a higher knowledge score (data not shown).

### Qualitative results

The main themes that emerged from analysis of the focus group discussions were: 1. *Perceptions and misperceptions of hypertension*, describing what the community members know and what they think they know about hypertension; 2. *Hypertension prevention and management challenges on community level,* describing to which extent the community is informed about hypertension and also describing the difficulties that are faced when looking for help, 3. *Recommendations to raise awareness about hypertension in the community,* describing the ways that awareness could be raised according to the community members. Data were further grouped into sub-themes as described below.

#### Perceptions and misperceptions of hypertension

Due to unfamiliarity with the term hypertension, in 5 out of the 6 FGDs the facilitators had to inform the groups that he/she was referring to “high high” or high blood pressure (as known in the community), to start eliciting the discussion. In the one English mixed group participants were not able to define hypertension except as “a sort of stress”, the other groups could identify and define high blood pressure.

Views on the causes and symptoms of hypertension shared by the participants were categorized into perceptions and misperceptions on the causes of hypertension, symptoms of hypertension, the physical appearance of a hypertensive person and what can be done to prevent and/or manage hypertension.

##### Perceptions and misperceptions on the causes of hypertension

Similar to the results in Table 2 focus group discussants cited salt intake, fast food (fatty foods), stress and alcohol as the most influential factors on the risk of getting high blood pressure. While salt intake was repeatedly mentioned as one of the main causes of hypertension, multiple participants specified that this only referred to the salt that was added to the food after cooking.

*The salt that I add to the food after it’s cooked is not good and sometimes you might put too much and you realize that he starts sweating white sweat because of too much salt. (isiZulu male FGD discussant)*

Stress/anxiety related factors prompting the development of hypertension revolved around the family situation and included fighting with children or spouse or not being able to provide for the family because of unemployment.

*Yes, stress also causes high blood pressure especially if you are not working, you have a wife and kids, the kids are crying asking for this and that, and the wife is yelling at you. (isiZulu male FGD discussant)*

*Most woman cause men to have high blood pressure. Communication is fundamental in a relationship, and things like failure to communicate, non-compromising attitude from woman and lack of respect results to high blood pressure. (isiZulu mixed FGD discussant)*

Across all the discussions there has been cognizance of the role of a healthy diet in hypertension prevention and the lack thereof within their community. Two distinct reasons for this were explained. The first common factor was poverty, since healthy living was not affordable to the majority, e.g. larger quantities of less healthy products can be purchased for the same cost as smaller quantities of the healthier option substitutes.

*…most of our communities, they can ill afford a healthy diet because it is expensive. Olive oil, 750 millilitres for 150 ZAR or it is not even 1 litre for 150 ZAR. You can buy sunflower oil 40 ZAR for 2 litre… (English male FGD discussant)*

*Poverty, because of the past, because ever since apartheid isn’t it that we are still economically deprived. Can’t afford healthy food, it is too expensive – healthy food. If you are supposed to have a few health foods per day it is expected to have seven colour vegetables and stuff. We really can’t afford, even the middle class people, they will do that on the Sundays but you’re advised as a participant of everyday. We can’t afford that.*

*(English male FGD discussant)*

On the other end of the spectrum there was a group who believed that even in the absence of poverty, people will still maintain unhealthy eating patterns.

*You know I remember when growing up we didn’t eat fats too much. Now they put oil into the chicken, pouring oil and braai it. We used to eat chicken boiled with water put salt and eat in the house. There was no high blood. You see we used to cook chicken, eat it, drink water and became right. Now they fry chicken and take cold drinks and drink cold drinks mixed with fats then the high blood goes up. We are no longer eating healthy food at first. There is no longer wholesome (margarine). We used to put wholesum on bread and eat, take cabbage and eat it and there was no high blood. Now it is fish oil and spices. You see those are the things that causes high blood - the food that we are eating. (isiZulu mixed group FGD discussant)*

Lastly, genetic predisposition to hypertension was debated. While some participants did not understand what hereditary meant, another group felt that they will become hypertensive despite any preventative efforts if it occurred in their family. The last group felt that a family history of hypertension is not a guarantee that all family members will be hypertensive.

*It’s in our blood, so it’s inevitable that I’m going to have it since it’s hereditary. (English male FGD discussant)*

*No, high blood pressure is not hereditary. My mother does not have high blood pressure but I have. (isiZulu Female FGD discussant)*

##### Perceptions and misperceptions on symptoms of hypertension

Nearly 90 % of participants (89.5% women, 89.2% men) answered that hypertension “makes you feel sick” (see Table 2). FDG participants elaborated on this by mentioning numerous perceived symptoms of hypertension, for example: headache, dizziness, sweating, swelling of the body, tiredness, loss of vision and weakness in the body.

*I think it differs with an individual, others will complain about severe headache like you know my veins are swollen and then when veins are swollen it means you have a headache and then another person will say I’m feeling drowsy time and again, I’m feeling sleepy. Another will say I did not feel anything, I just felt dizzy and when got to the clinic, was told that high blood had increased… (English mixed FGD discussant)*

*Unless you are ignorant, the symptoms of high blood pressure are easily identified. You see them really well when the person is angry, you will see a person sweating but its symptoms are visible and easy to identify. (isiZulu mixed FGD discussant)*

When talking about the way a hypertensive person looks like, most participants agreed that you cannot see if a person has hypertension based on physical appearance.

*Yes because my mother is somebody with high blood pressure, but she’s looking healthy.... (English female FGD discussant)*

##### Perceptions and misperceptions on how to prevent/manage hypertension on an individual level

Exercising and eating healthy were predominantly brought forward as ways to improve high blood pressure. Other things that the participants shared were: avoiding stress, maintaining a healthy body mass (i.e. no obesity), no alcohol, going to the clinic and adherence to medication.

*To exercise, to eat well, to eat healthy food and veggie and fruits, like you eat banana, apple, all the fruits and all the veggies like spinach and spinach. (English female FGD discussant)*

*It is important to take treatment because it shows that you want to get better and you are also considering your family not to worry them with any complications from the condition. But if you decide to stop treatment, you are not only hurting or killing yourself but you are also hurting your family. (isiZulu mixed FGD discussant)*

Men and women believed that hypertension and/or stress reduction could be managed by going to church and/or praying, reflecting the deep trust and respect these community members have for their faith and for its ability to heal.

*For us to defeat high blood pressure, I think we should also attend church. Church will provide us with inner peace and help us not to think too much and also reduce stress from debts, unemployment. (isiZulu male FGD discussant)*

#### Hypertension prevention and management challenges on community level

The participants mentioned various challenges for dealing with hypertension in the community. These challenges were organized into two categories: the knowledge of the community members and challenges in seeking health care.

##### Views on the knowledge of the community members

The predominant view was that knowledge about the disease and how to treat it comes with a diagnosis.

*Those who have been diagnosed with high blood pressure know about it however those who do not have high blood pressure are also not well informed. (isiZulu mixed FGD discussant)*

*Most people do not know about high blood pressure and are only likely to know once it has been detected or diagnosed. They do not know how to prevent it and most people will talk about it after diagnosis and that is when you told to change your diet like stop eating food with too much salt and cooking oil. (isiZulu male FGD discussant)*

##### Challenges in seeking health

The primary factors affecting health-seeking behaviour were related to the health system, specifically health care worker attitudes, waiting times at the clinic and distance to the clinic.

*Sometimes you will find that a nurse assisting you has her personal issues as well and she takes her problems out on you. This other time, I had to intervene when a nurse was shouting at an old woman who probably was at her mother’s age. Nurse struggle to put their personal matters aside when seeing patients. (isiZulu male FGD discussant)*

*Waiting times are a problem, sometimes you wait long times and health workers would on a tea break, and lunch while you are still waiting. (isiZulu female FGD discussant)*

*Yes distance is a problem and sometimes you have to take public transport and you probably do not have money. You end up not going to clinic because of distance and travelling cost to the clinic. Sometimes when you get there you are already tired because of the distance. (isiZulu male FGD discussant)*

The second factor affecting health-seeking behaviour was the lack of intrinsic motivation to seek help.

*I think I said it, I’ll repeat it. I think its laziness…. (English male FGD discussant)*

#### Recommendations for raising awareness in the community about hypertension

Almost all community members agreed that efforts should be increased to raise awareness about hypertension in the communities surrounding Elandsdoorn. Some ideas were mentioned in multiple or all discussions, while others were only raised in one or two focus groups but are still noteworthy enough to include.

##### Door-to-door care vs. visiting the clinic

Door-to-door visits for hypertension awareness and diagnosis were strongly recommended, similar to the way home-based HIV-testing is conducted [20–22]. The most important reason given was that with this method community members will be in a comfortable and safe space and the health care sessions can take place free of stigma related challenges.

*But if you go to my place, you knock at the door, you say, you ask me: do you have time, I’m only asking for 10 minutes of your time. Then I’m going to grant you that 10 minutes, then you’re quite sure that in that 10 minutes period of time I’ve learned something. (English mixed FGD discussant, female)*

*I prefer door to door because some people are lazy to go to the clinic, so door to door I think is the best way. (English female FGD discussant)*

However, not everybody agreed with this, fearing stigmatization as a result of home visits.

*Coming to my house will make it easy for health workers to discuss my privacy if they discover that I am sick. They would easily talk about me. (isiZulu female FGD discussant)*

##### Ways of raising awareness in the community

The main way of raising awareness mentioned by participants was education through peer educators, which is done by targeting and educating the younger generation who in turn can educate the older generation.

*Education is the key, so that they can be able to get the message through and then by initiating that we can start at school. In the local primaries, educate our children, and then therefore they’ll go to the community back and say: mom I have some assignment, can you help me what is hypertension. (English mixed FGD discussant)*

Noting the respect for the church in the community and the possible coverage, several participants recommended working with church leaders as a mechanism to increase awareness of hypertension.

*What I think could be done, is to find those leaders in those beliefs and be taught about those things so that when they get to, let’s say, for example in churches, they’ll be able to talk about it you know. (English male FGD discussant)*

Other ways that were mentioned included raising awareness through (sporting) events or through community dialogues, where subjects like hypertension could be discussed.

*… the doctors and nurses and professionals and those one who know about this things come and tell about this thing. Come and share information in a funny way, in a fun way that even young people will be interested to come. (English male FGD discussant)*

Moreover, mobile clinics with incentives, pamphlets and billboards were proposed as effective methods of disseminating information. The suggestion of an incentive incited mixed reactions. Some of the participants felt that incentives would divert the community members from the healthcare workers’ health promotion efforts (particularly as they related to health messaging and increasing patient responsibility and accountability for their own health and wellbeing).

*So in most cases, when there is a give-a-way, there is no learning there, because I’m expecting what I am going to get back instead of what I am going to hear so I really don’t like the incentive part of it. (English mixed FGD discussant, female)*

## Discussion

This study aimed to investigate knowledge of and perceptions about hypertension of community members residing in a low-resource rural area in South Africa. In addition, it assessed how community awareness could be raised for hypertension. Overall, there was congruence between questionnaire and focus group discussion data and results of focus group discussions were similar for both men and women irrespective of language.

Three main themes emerged from the data: p*erceptions and misperceptions of hypertension*; *hypertension prevention and management challenges on community level*; and *recommendations for raising awareness in the community about hypertension.*

Most of our population seemed to have intermediate or good knowledge of hypertension, and only 11.8% had poor knowledge. Perception of, respectively knowledge about hypertension reported in our and previous studies seems to be very well aligned; previous research in a rural South African hospital [18] reported psychological stress and fatty/unhealthy food as the primary contributors to hypertension development. Moreover, participants were generally well informed about the ways to reduce the risks for hypertension, citing most often a healthy diet, physical exercise and taking treatment in line with previous research studies conducted in South Africa [15, 16] and elsewhere [23, 24]. Although general knowledge on major risk factors for hypertension was present in this community, they felt that poverty limited their ability to choose healthy lifestyles such as nutritious foods and recreational physical activity. Because the prevalence of hypertension in South Africa continues to increase there is an urgent need to improve health awareness and to address social determinants of health supporting healthy lifestyle choices.

The main misperception that came forward is that hypertension results in symptoms like headache, dizziness and sweating; a finding previously reported in rural populations in South Africa [17, 18] as well as in multiple studies conducted in both high and low-income countries [24, 25]. Targeting this misperception as part of intervention strategies by educating more people about the dangers of hypertension as a silent killer could potentially increase the percentage of people participating in blood pressure screening activities.

On a community level, several challenges for seeking health care were mentioned for both prevention and treatment including limited means, i.e. to afford public transport to get to clinics for blood pressure management and health care facility factors such as health care worker attitude, long waiting times at the clinic as well as lack of intrinsic motivation to seek help. Another challenge brought forward was that knowledge about hypertension only comes after diagnosis for most members of the community.

The most prominent recommendation to raise hypertension awareness was door-to-door visits, where health care workers visit the community members to do home-based blood pressure measurements and give information on prevention and management of hypertension. This intervention strategy has been shown to be highly effective in raising HIV awareness in both South Africa [22] and other countries in sub-Saharan Africa [20, 21].

Our findings highlight the need for campaigns that focus on ways to create a healthy diet and lifestyle that fits a low income. For instance, campaigns could aim at showing the benefits of reducing salt and fast food and teach people to choose healthier food alternatives such as vegetable oils (like sunflower oil), fresh fruits and vegetables. Health campaigns could come in a format attracting a younger audience, such as events where the people are educated in a way that is fun to them. Awareness could also be raised through home-based sessions and by collaborating with church leaders.

### Strengths and limitations

To the best of our knowledge this is the first comprehensive mixed methods study on the perception of hypertension in rural South Africa.

However, this study is not without limitations. All participants included in this study are part of an on-going cohort study, for which they need to come to the study centre on a yearly basis for check-ups. This population might be more aware of health issues such as hypertension due to the regular visits, and may therefore not be representative of the general population. As participants were not all fluent in English, counsellors assisted in isiZulu or Sepedi which could have led to additional probing by the counsellors, influencing participants’ responses. Lastly, this study was conducted in a rural geographic location in South Africa, limiting generalizability of the findings to (peri-)urban settings in South Africa and other sub-Saharan Africa countries.

## Conclusion

This study provides useful insight for healthcare programmers on hypertension knowledge and perception in a rural community to be used to develop and shape community level hypertension prevention and management interventions. It further highlights the need for improved health promotion efforts to increase knowledge of hypertension in rural communities, and to address poverty as a major obstacle to support healthy life-style choices.

## Additional files

Additional file 1: Hypertension perception questionnaire. This is the questionnaire used for the quantitative part in this study (PDF 44 kb)

Additional file 2: Study guids focus group discussions. This is the study guide used by the moderators during the focus group discussions (PDF 67 kb)

## Abbreviations

CVD: cardiovascular disease
FGD: focus group discussion
NCD: non-communicable diseases
NCS: Ndlovu cohort study
WHO: World Health Organization
