# Supplementary material for: Hypertension in a rural community in South Africa: what they know, what they think they know and what they recommend
Source: BMC Public Health. 2019 Mar 25;19:341. doi: 10.1186/s12889-019-6642-3 (PMC6434617; doi:10.1186/s12889-019-6642-3)
Supplement: Supplementary file 1 — Hypertension perception questionnaire. This is the questionnaire used for the quantitative part in this study (PDF 44 kb) [file 12889_2019_6642_MOESM1_ESM.pdf]

Hypertension perception questionnaire

Study ID ______________________

Date ______________________

Heart study ID Number ______________________
(Heart study ID)

Qx1 Have you ever been informed by a medical practitioner or nurse that you have high blood pressure?
- ◯ yes
- ◯ no
- ◯ don't know

Qx2 Are you taking medication for your high blood pressure?
- ◯ yes
- ◯ no
- ◯ don't know

(if yes for qx1)

Qx2a How often do you take your treatment?
- ◯ Daily
- ◯ 5-7 days a week
- ◯ 3-5 days a week
- ◯ 1-3 days a week
- ◯ 1 day a week
- ◯ less often
- ◯ don't know

Qx3 Has a medical practitioner or a nurse discussed life style changes (e.g. changing your diet, quit smoking, exercise more) with you?
- ◯ yes, please specify
- ◯ no
- ◯ don't know

(if yes for qx1)

Life style changes advised ______________________

Qx3a Do you adhere to the life style advice / changes?
- ◯ yes fully
- ◯ yes partly
- ◯ no
- ◯ don't know

(if yes for qx3)

Qx4 Do you think your chances of getting a disease of your heart or blood vessels (like stroke or a heart attack) are
- ◯ very high
- ◯ high
- ◯ moderate
- ◯ low
- ◯ very low
- ◯ don't know

Qx5 Has anyone in your direct family ever been diagnosed with high blood pressure?
- ☐ Mother
- ☐ Father
- ☐ Sister, please specify how many
- ☐ Brother, please specify how many
- ☐ Daughter, please specify how many
- ☐ Son, please specify how many
- ☐ Grandmother
- ☐ Grandfather
- ☐ Grandchildren, please specify how many
- ☐ No one
- ☐ Don't know

Direct family with high blood pressure

____

Qx6 Do you know anyone in your surrounding, other than your family, who has been diagnosed with high blood pressure?

- ◯ yes (please specify)
- ◯ no
- ◯ don't know

People with HBP in surroundings

____

Qx7 How well informed do you think you are about high blood pressure?

- ◯ very well
- ◯ well
- ◯ moderate
- ◯ low
- ◯ very low
- ◯ don't know

Qx8 Do you think the following factors influence the risk of getting high blood pressure?

	yes	no	don't know
Physical exercise	◯	◯	◯
Smoking	◯	◯	◯
Diet	◯	◯	◯
Stress	◯	◯	◯
Religion	◯	◯	◯
Family History	◯	◯	◯
Alcohol Consumption	◯	◯	◯
Gender	◯	◯	◯
The weather	◯	◯	◯
Salt Intake	◯	◯	◯
Pregnancy	◯	◯	◯

Qx9 Are the following statements true or false?

	true	false
Having high blood pressure is bad for you	◯	◯
Frequent consumption of fast food (like KFC) can result in an increase in your blood pressure	◯	◯
Kitchen salt (sodium) increases your blood pressure	◯	◯
Drinking of alcohol increases your blood pressure	◯	◯
Blood pressure gets better by a healthy diet	◯	◯
Blood pressure gets better by exercise	◯	◯
Blood pressure can be managed by going to church	◯	◯
Blood pressure can be managed by praying	◯	◯
Blood pressure can be managed by going to the traditional healer	◯	◯
High blood pressure makes you feel sick	◯	◯
High blood pressure is a rare condition	◯	◯
High blood pressure prevents the development of heart disease	◯	◯
High blood pressure increases the risk of a stroke	◯	◯
If you ignore it, high blood pressure will go away on its own	◯	◯
