# Supplementary material for: Hypertension in a rural community in South Africa: what they know, what they think they know and what they recommend
Source: BMC Public Health. 2019 Mar 25;19:341. doi: 10.1186/s12889-019-6642-3 (PMC6434617; doi:10.1186/s12889-019-6642-3)
Supplement: Supplementary file 2 — Study guids focus group discussions. This is the study guide used by the moderators during the focus group discussions (PDF 67 kb) [file 12889_2019_6642_MOESM2_ESM.pdf]

Study guide focus group discussion

Introduction: People suffer from different conditions of the heart and of the brain. Examples of these conditions include stroke (which leads to paralysis and loss of feeling to one side of the body), heart disease (which is characterized by dull, pressing pain of left side of the chest where the heart is located while carrying out heavy tasks), heart attack (which is usually abrupt and sometimes leads to death), but also hypertension (high blood pressure) and diabetes (high blood sugar). In this discussion group we will primarily focus on high blood pressure. We will discuss about your knowledge of and experience with this condition, in order to understand the condition better. We will seek your views on how hypertension is caused and how it can be prevented. One of the researchers joining the focus group discussion, will moderate the discussion while the other researcher takes notes. Everybody is invited to join the focus group discussion, because we are interested in what you think about the subjects. Please remember that there are no wrong answers!

Moderator to start with an icebreaker:

You will remember when we explained the study to you, you signed a form giving permission to record the discussion. When we leave here the discussion will be typed up into a document. We will not be able to identify who is saying what when we are typing up the transcript. Therefore before we start I am going to give each of you a letter of the alphabet – starting with A on my right. Each time before you speak you will need to say your letter. Don’t worry before we start the actual discussion we will practice.

Topic areas to be discussed:

1. Let us talk about which lifestyles and behaviours can cause someone to get high blood pressure.
   Probe: Alcohol, smoking, diet, etc.
   Probe how they think that those factors contribute to hypertension

2. Apart from the lifestyle choices and behaviours, are there other factors that can cause an individual to be at risk of developing high blood pressure?
   Probe: marriage, number of children, living arrangements, poverty/income, other illnesses.
   Probe how they think that those factors contribute to hypertension

3. Do you think that it hypertension is a problem?
   Probe whether they think it is serious/ life threatening
   Probe what their thoughts are on how many people have it in the community, world

4. How do you think high blood pressure can be avoided, prevented?

   a. *Please describe what can be done to prevent someone from acquiring any of the conditions described above?*

5. What kind of complaints do you think people with high blood pressure have?
   Probe what they think a hypertensive person looks like

6. What do you think the community members know about high blood pressure and how to prevent it?

7. What can be done to raise awareness of such diseases in the community?
   Probe whether they think that more needs to be done
   Probe how they think the awareness and testing can be done for easier access (example door to door versus coming into the clinic)

8. Who should go for blood pressure measurements?

9. How often should your blood pressure be tested?

10. What makes it difficult to access and utilize care/treatment for the conditions described above?
    Probe: Healthcare worker attitudes, cost, fear of checking, having time to go to the clinic to get medication
